# Supplementary material for: LncRNA 220: A Novel Long Non-Coding RNA Regulates Autophagy and Apoptosis in Kupffer Cells via the miR-5101/PI3K/AKT/mTOR Axis in LPS-Induced Endotoxemic Liver Injury in Mice
Source: Int J Mol Sci. 2023 Jul 7;24(13):11210. doi: 10.3390/ijms241311210 (PMC10342868; doi:10.3390/ijms241311210)
Supplement: Supplementary file 1 [file ijms-24-11210-s001.zip › Original images (H & E, WB, IF, Flow, and TUNEL).pdf]

Figure 1A

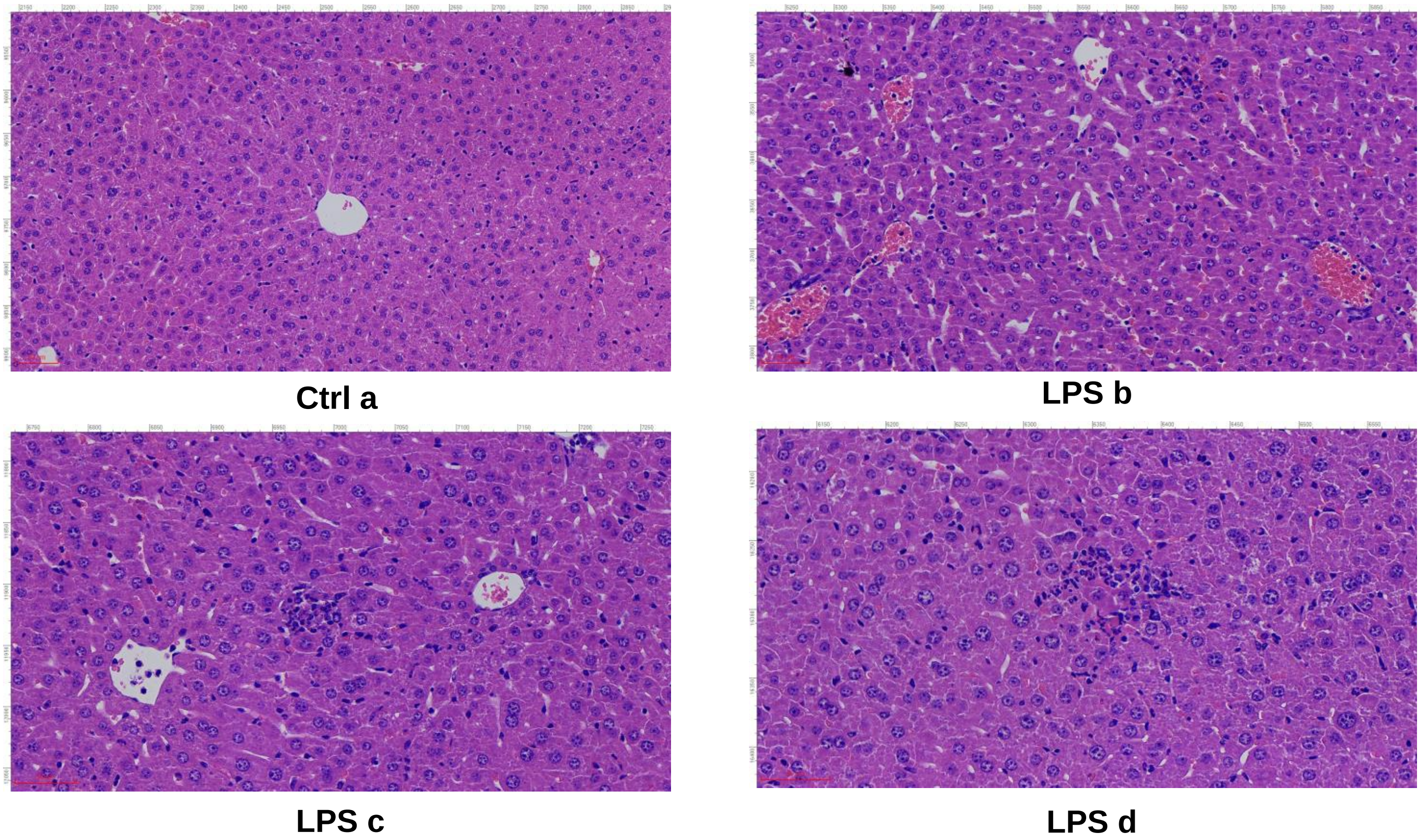

Figure 2A

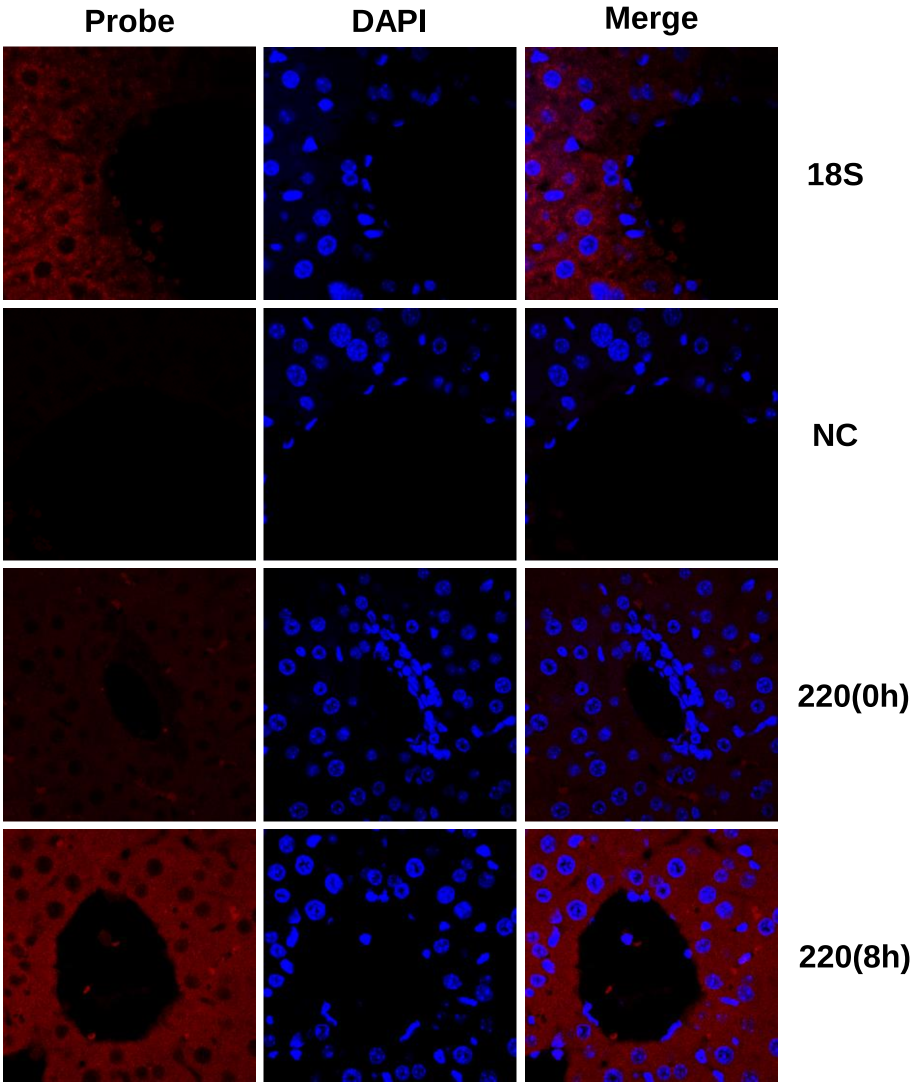

Figure 2F

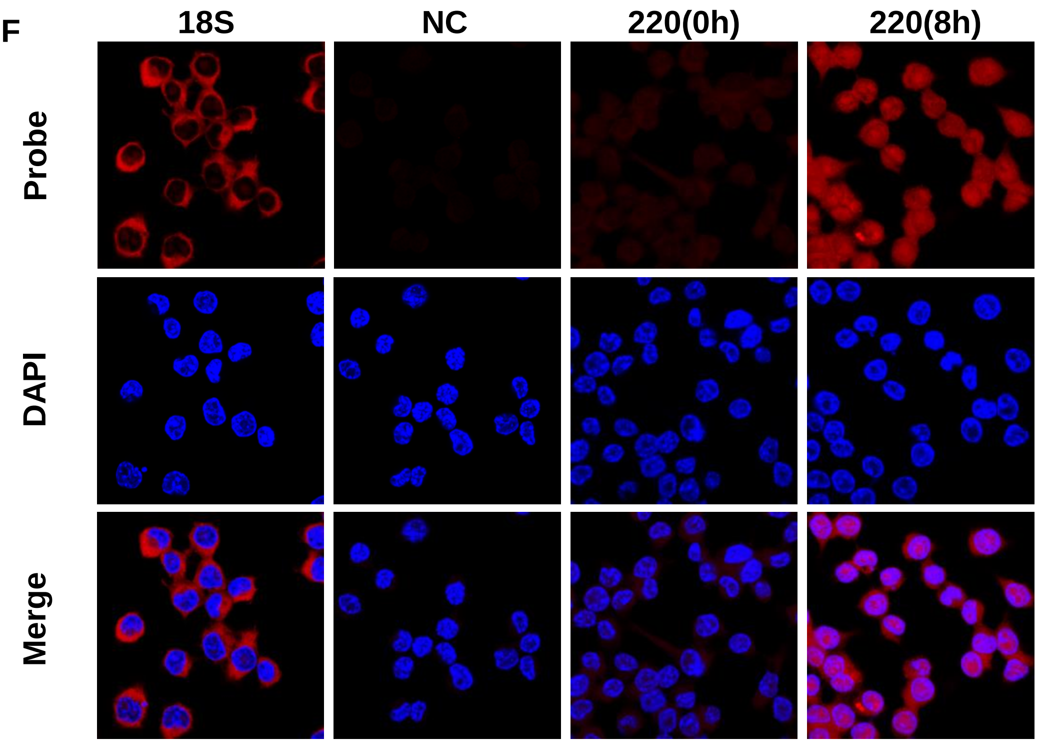

Figure 5A

WB results after overexpression of 5101

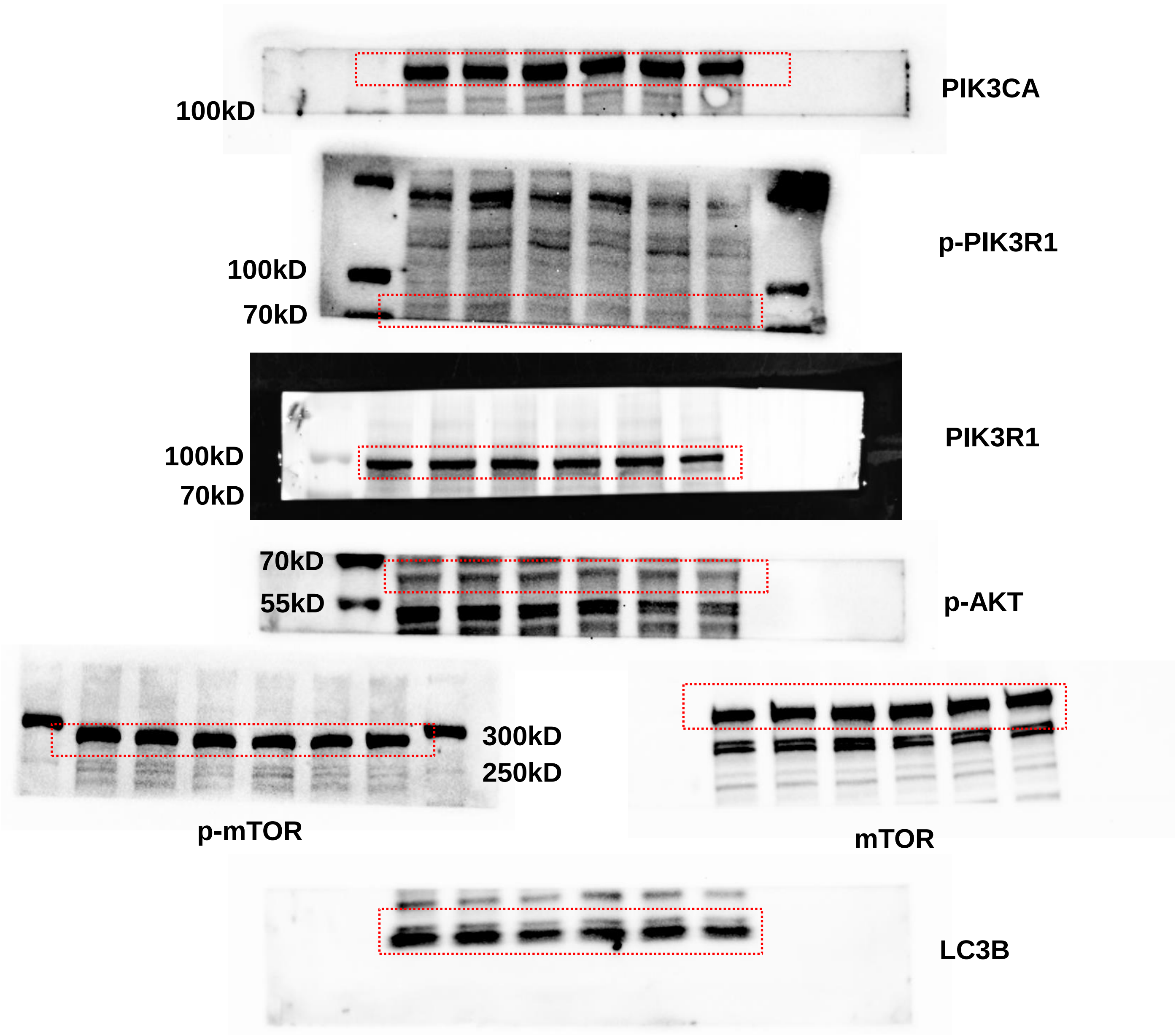

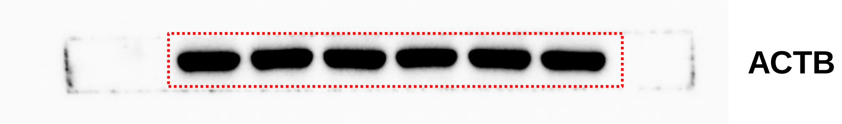

Figure 5C

WB results after knockdown of 5101

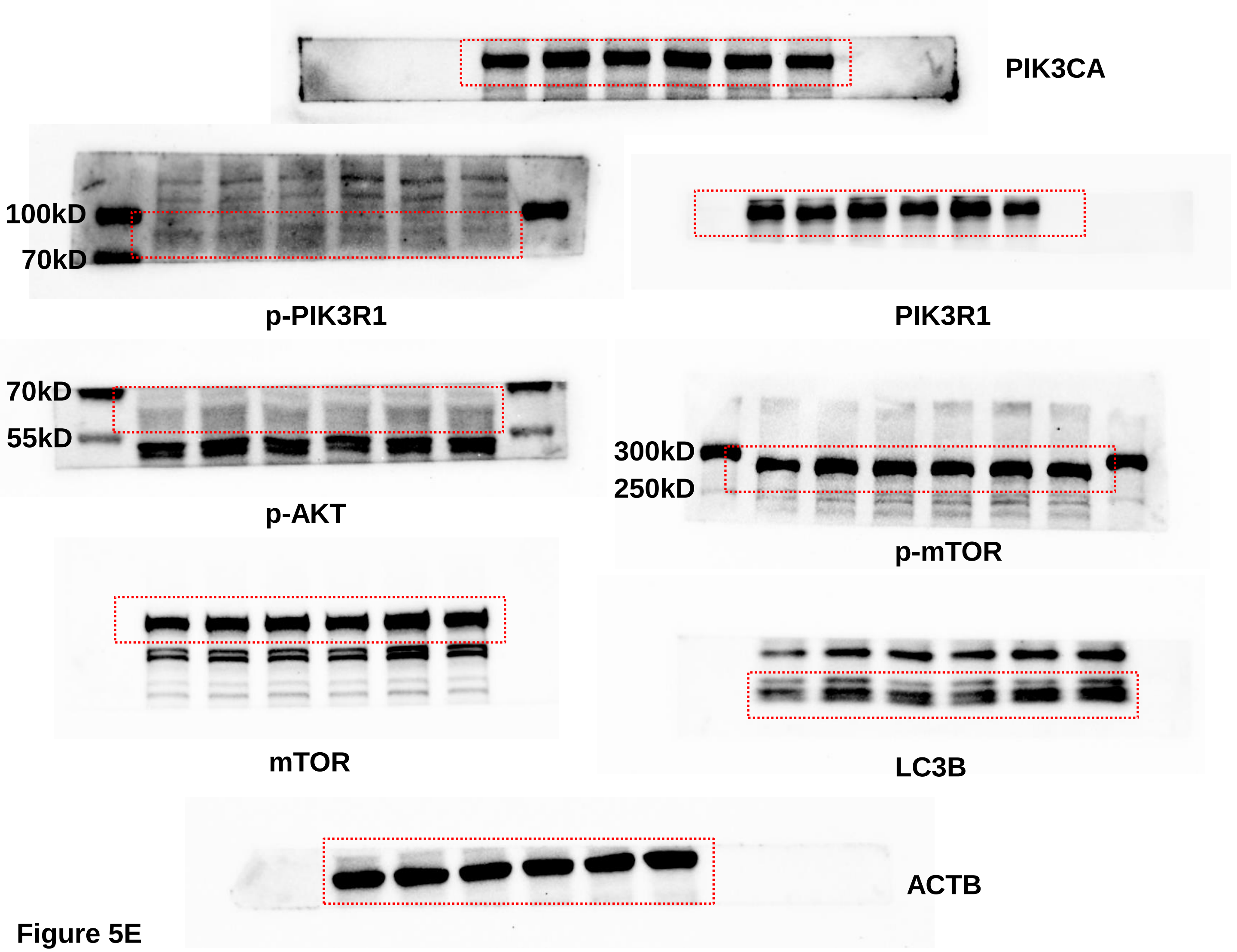

Figure 5E

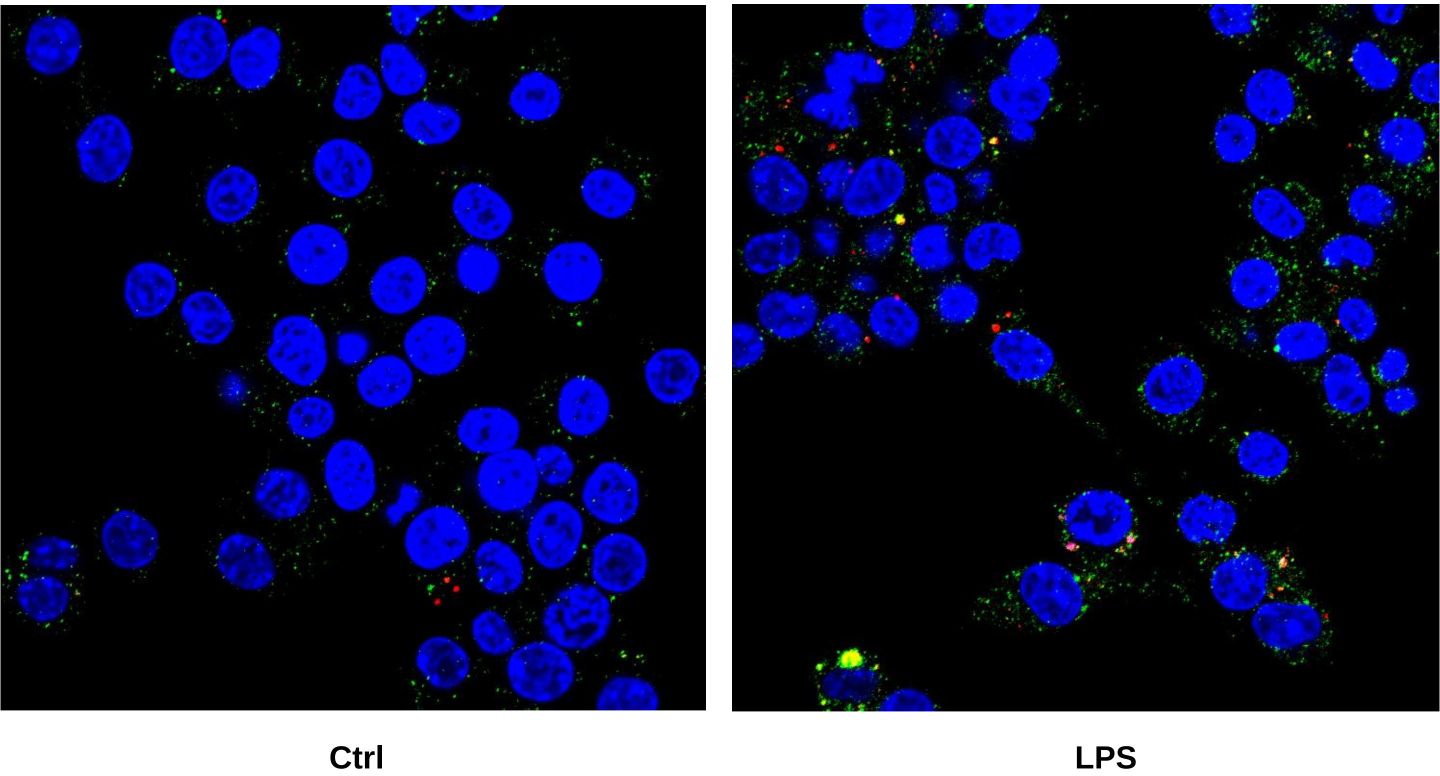

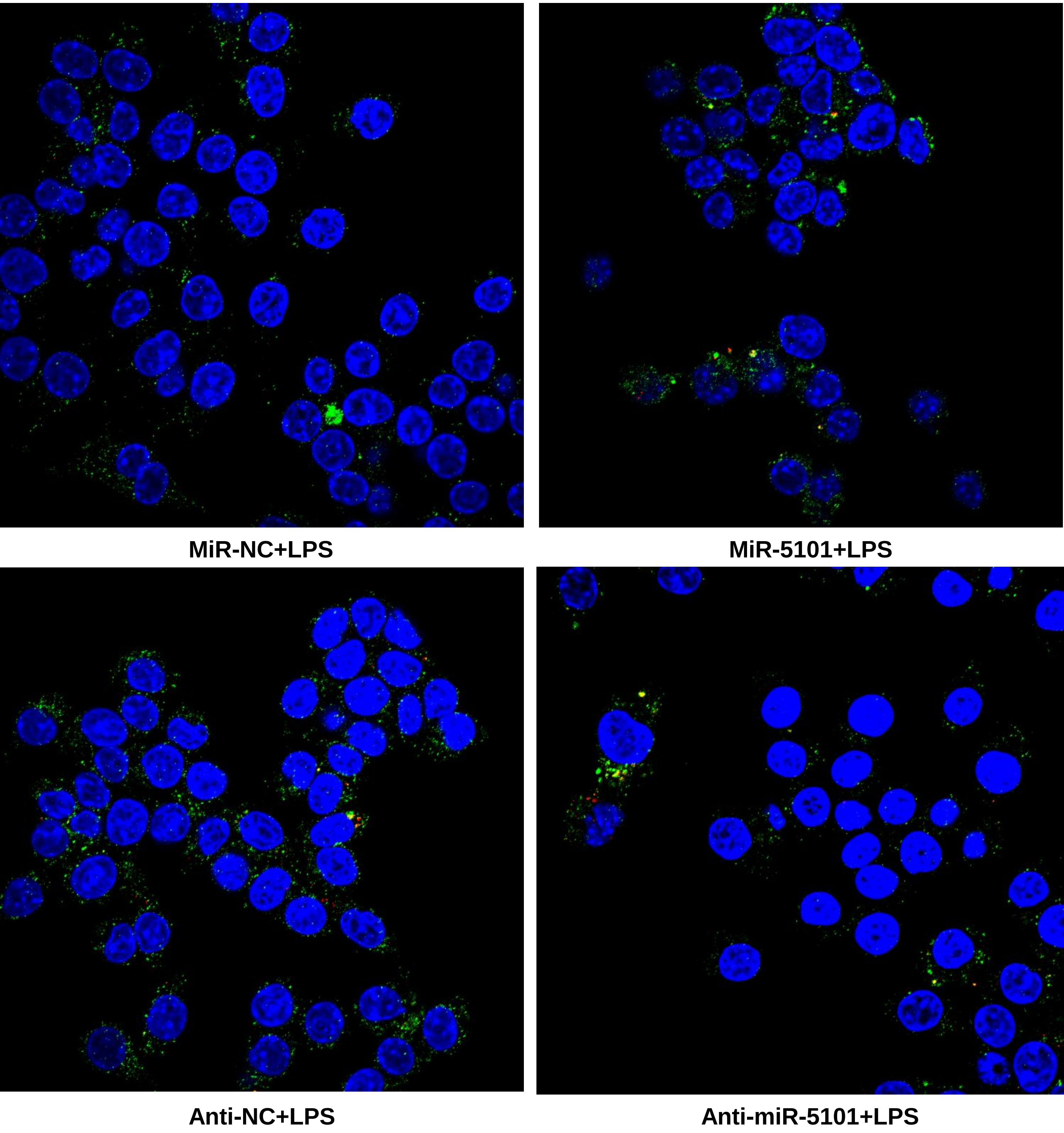

Figure 6A

WB results after overexpression of 5101

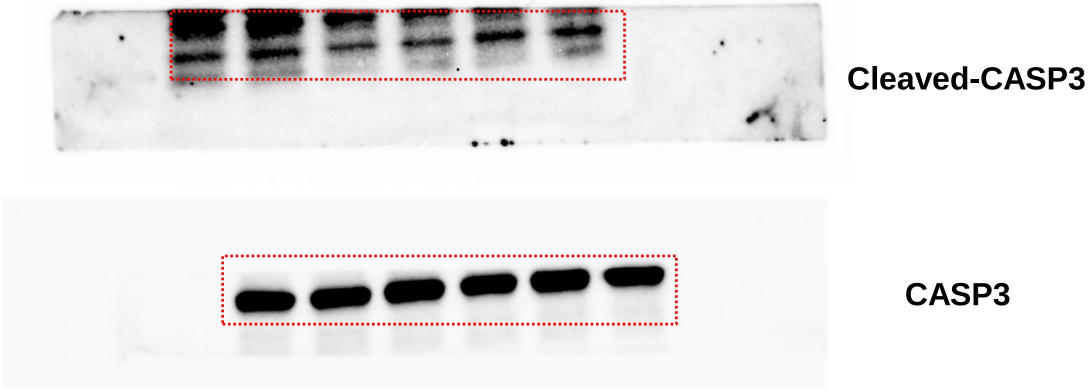

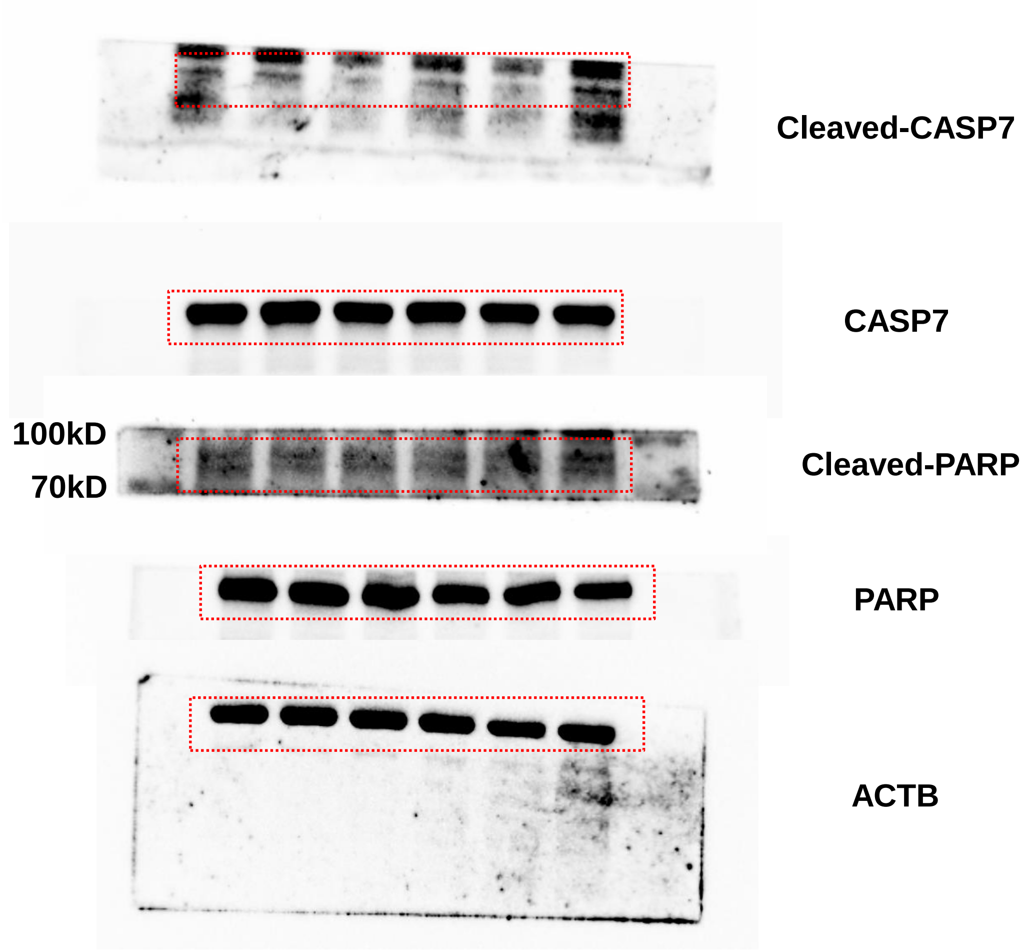

Figure 6C

WB results after knockdown of 5101

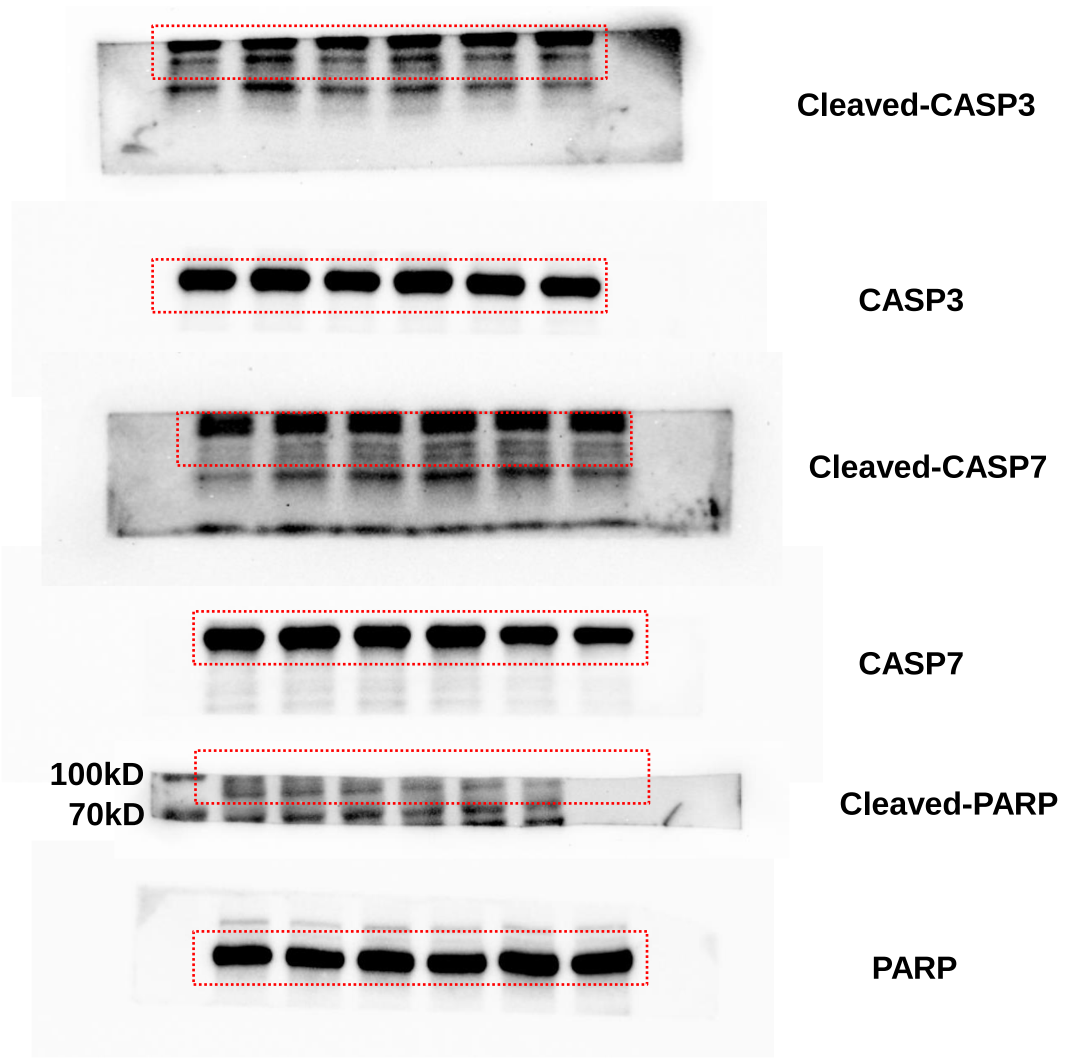

Figure 6E

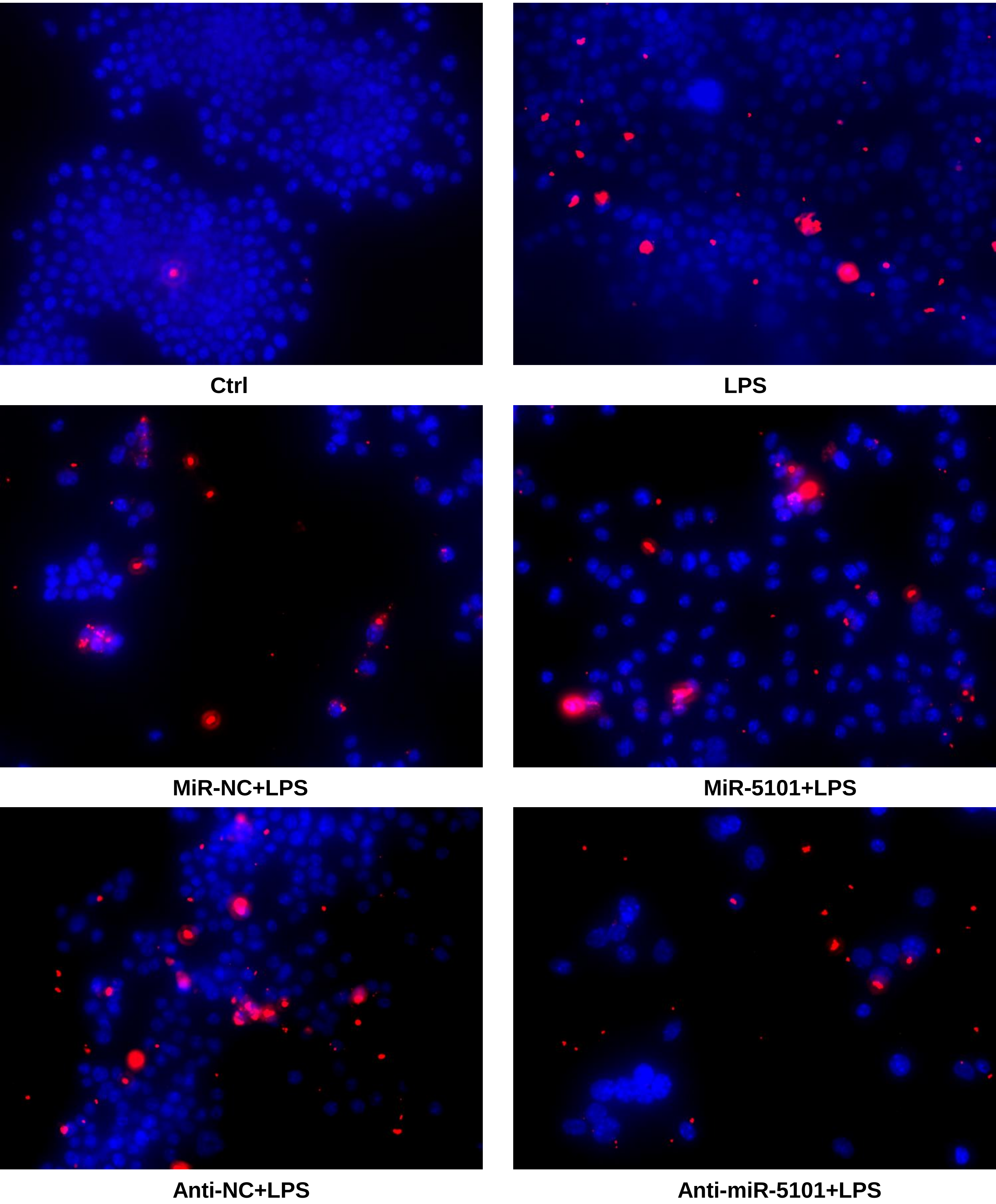

Figure 6G

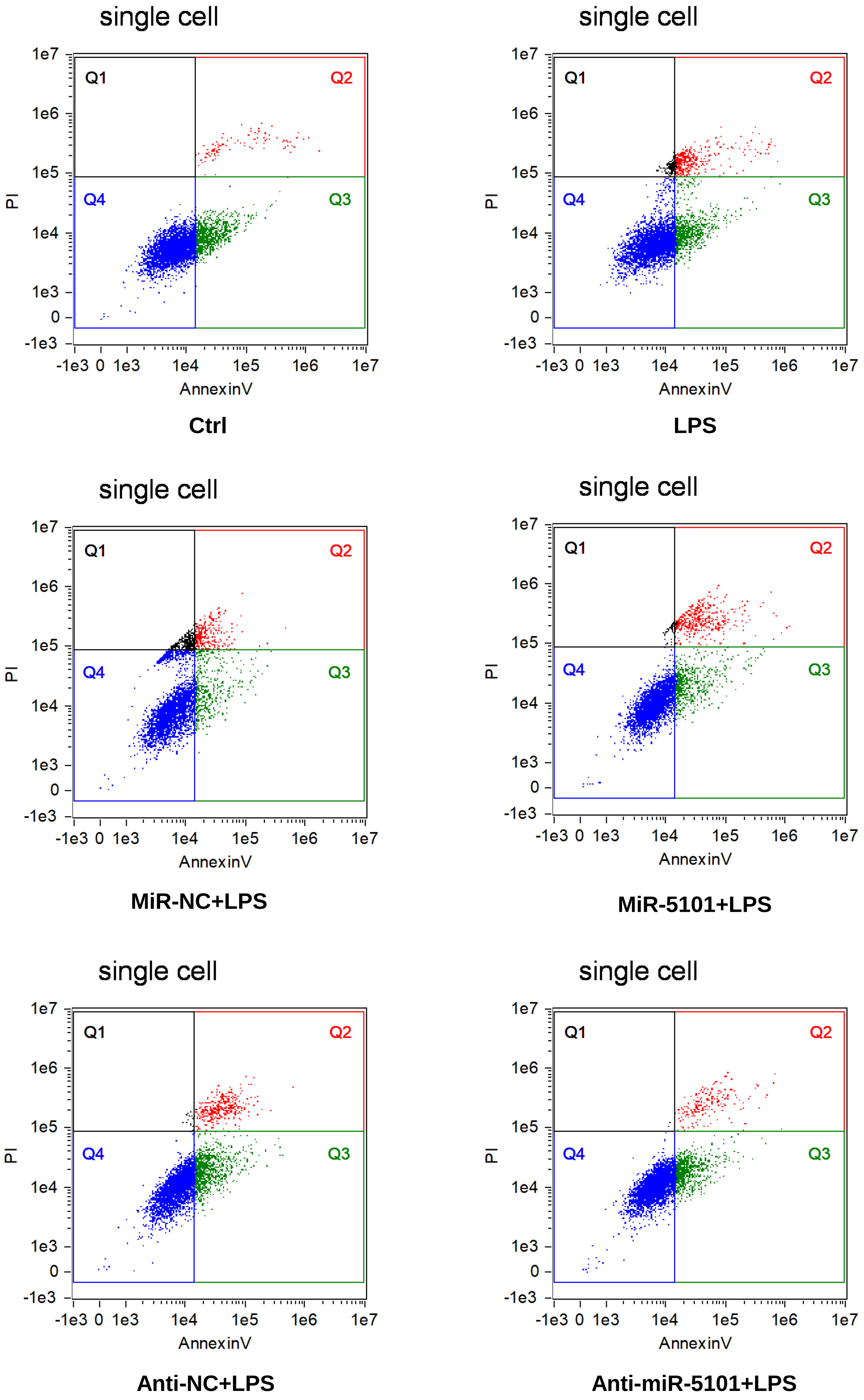
single cell
Q1
Q2
Q3
Q4
1e7
1e6
1e5
1e4
1e3
0
-1e3
PI
-1e3 0 1e3 1e4 1e5 1e6 1e7
AnnexinV
Ctrl
LPS
MiR-NC+LPS
MiR-5101+LPS
Anti-NC+LPS
Anti-miR-5101+LPS

Figure S2B

WB results after overexpression of 5101

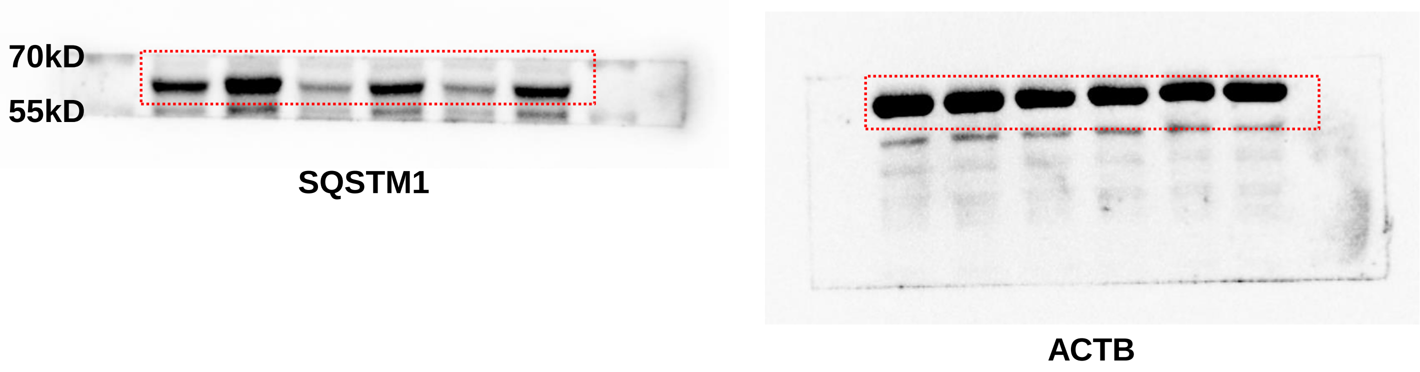

Figure S2D

WB results after knockdown of 5101

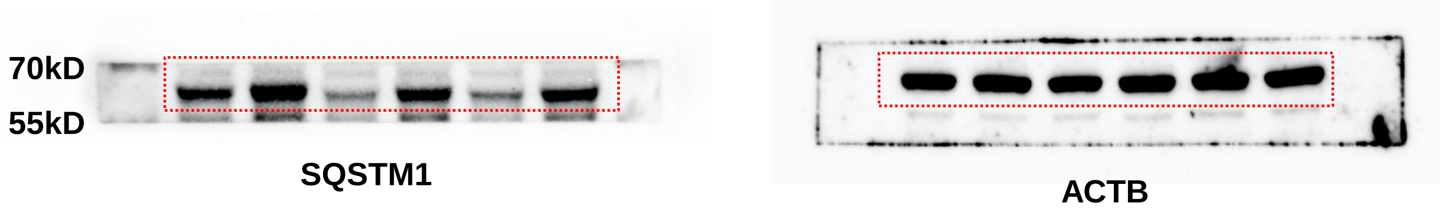

Figure S2F

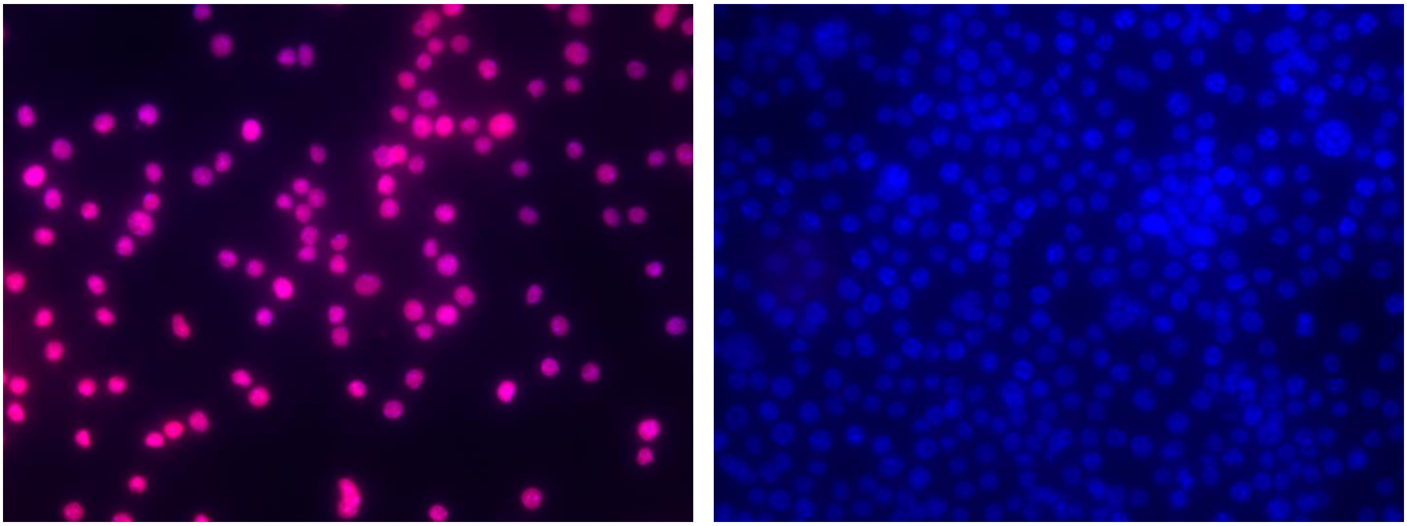

Positive control **Negative control**
