# Supplementary material for: LncRNA 220: A Novel Long Non-Coding RNA Regulates Autophagy and Apoptosis in Kupffer Cells via the miR-5101/PI3K/AKT/mTOR Axis in LPS-Induced Endotoxemic Liver Injury in Mice
Source: Int J Mol Sci. 2023 Jul 7;24(13):11210. doi: 10.3390/ijms241311210 (PMC10342868; doi:10.3390/ijms241311210)
Supplement: Supplementary file 1 [file ijms-24-11210-s001.zip › Sequence of 220.pdf]

>TCONS_00127483 11 5488251-5507220
AGAGACAAGGAAACAGGCTCTGAGAGATTAAGCAACTCATCCCACTTGCTCAACCAGCATATGTGGGAG
ACCTGACAACCCGCCCTGTGTGGCCGTTTCACAAGCCAACGCCCAGCCACAGGGGACGACTGTTCCTGG
GACTGGAAGTGATGAAGAGGAGCCATGATGGTTAAACTTCTTTAAAAGAAAGAAGACAAACTGGCTTTC
CTTGATGCCTTTGAACCTGAGTCCAGAAGACTTAGGGACAATGGATAGCTAAAATTTATTTATATCTGA
AGTTGGGTGGCAGGGTGCACTCCAGAGGCAGAGGCAGAGGCAGGGGGATCTATCTCCGAGTCTGAGGTC
AGCCTGGTCTACAGATCGAGTTCCAAGACAGCCAGGGCTACAAAGAAAAATCCTGCCTCAAAAAATGGA
AAAAACAAAACAAAACAAACAAAATACATTAAAACAAACAAACAAACAAACAAACTGGATTTATTCTCT
GCCGCGGCTGGGAGTGGACACCTTTGTTCAGCAAAATGAACCTTTTCCTGATTGTGCACTTCCAGGCCC
TGGGCAAGCCGACTCTAGCACTCTGCAAAACCCCACCTCCCAAAGGAGGGTTGAACTTCTTGGAACCCT
TAAAAACTACCAGTGAACTTCAGGAAGTAACCCACGCAACTCTTTCCCAGAAGAGGTCAATGGTCACAG
AATCGTAAGAAACACCACTCTGACCCCGGGACTTCCTGCCTCCACACTCGGAACATCTCAAAGTTCAAA
CTGCTCAGGAACTGACATCCGGCTGACAGACGGGGAGTGTCGCTCAGTGGTAGAGTGCTTTCCTACATC
GGGAGAGGCCCTGGCTCCTATCTCCAACTCTGTAAAGAAAAGATTAATACTATGAAACGAAAATAAACC
AGGCTTTAAAGAAAACAAAAGCATGAGAAGAAGAAATCGCTTTCCCTGGGGTCTGGTTCCGTCGTCGTC
CCCACCCCCGCCCCGCCCCCATGACTGAGTCAAAAGATTTCAATGCAACTGGTCAAACCAAGAGTGCCT
GCAGAGATCTTGCATGTTTTCCTAAAGCCATCAGACAAGAGTACAACAAAGAATGGGCAGATGTGCTTA
CTCAGGACCAGAACACCTCTGACCGATTAGCATTCCTCTCTTAACCCCGCCCCCCCACCCCCCCCATGT
AAACAGTTTCACCTCATAAAACCAGGTTTTTTGTTTTTTTTAAAAAATATTTGGTTAAGGGGGCTGGTG
AGATGGCTCAGTGGGTAAGAGCACCCGACTGCTCTTCCAAAGGTCCGGAGTTCAAATCCCAGCAACCAC
ATGGTGGCTCACAACCATCCATAACAAGATCTGATGCCCTCTTCCGGAGTGTCTGAAGACAGCTACAGT
GTACTTACATATAATAAATAAATAAATAAATCTTTAAAAAAAAAATATTTGGTTAAAAAACAAATAAAA
CAAACAAACAAACAAACAAAAAAAGCAGACCAAAATCCAAAAAACTCTTGGGCTATCAAGATGGCTCAG
TGACTAAAATTACCTGTCACCAAGCCTGATCCATCCTCAGGTTGACCTTTAACCTCCACACTCACACCT
GCCATATGTACAGAAACATGCTCTCCAGCACACGCACACGTGCGCGCACACACACAACTAATAAACAAA
TAAATGCGATGCATTTTTAGAAGAACATTCTCAAAGACACGATAAACCCCAGGCTAATACATTTTACTA
ATTTTTTTTTTTTCTGAGACAGGGTTTCACTATGCGGCCCTAGCTGTCCTTAACTCACTCTGTAGACCA
GGCTAGCTTTGAACTCAGAAATCTGCCAGCCTCTGCCTCTCCAGTACTGGGACTAAAGGTGTGTGCCAC
CACCCCCTGGCTTTACTTATCTTTT
