# Supplementary material for: LncRNA 220: A Novel Long Non-Coding RNA Regulates Autophagy and Apoptosis in Kupffer Cells via the miR-5101/PI3K/AKT/mTOR Axis in LPS-Induced Endotoxemic Liver Injury in Mice
Source: Int J Mol Sci. 2023 Jul 7;24(13):11210. doi: 10.3390/ijms241311210 (PMC10342868; doi:10.3390/ijms241311210)
Supplement: Supplementary file 1 [file ijms-24-11210-s001.zip › Supplementary figure legend.pdf]

Supplementary figure legend

Figure S1. (A) Heat map of differentially expressive lncRNAs in the livers of mice stimulated by LPS at different timepoints (0 h, 2 h, 8 h, and 24 h). (B, C) Prediction of the subcellular distribution of 220.

Figure S2. (A) Screen-out of si-220. (B, C) WB results pertaining to the protein level of SQSTM1 after overexpression of 5101. (D, E) WB results pertaining to the protein level of SQSTM1 after knockdown of 5101. (F) Positive and negative controls of systematic detection for TUNEL assay. (G) Correlative heat map of various immune cells based on the results of immune cell infiltrative analysis. (*, $P<0.05$, ****, $P<0.0001$; ns, not significant)
