# Supplementary material for: LncRNA 220: A Novel Long Non-Coding RNA Regulates Autophagy and Apoptosis in Kupffer Cells via the miR-5101/PI3K/AKT/mTOR Axis in LPS-Induced Endotoxemic Liver Injury in Mice
Source: Int J Mol Sci. 2023 Jul 7;24(13):11210. doi: 10.3390/ijms241311210 (PMC10342868; doi:10.3390/ijms241311210)
Supplement: Supplementary file 1 [file ijms-24-11210-s001.zip › Supplementary Table.pdf]

Table S1 Primer sequences of qRT-PCR

Gene	Primer	Sequence
220	Forward	GGTCAAACCAAGAGTGCCTG
	Reverse	TCGGTCAGAGGTGTTCTGGT
18S	Forward	AGTCCCTGCCCTTTGTACACA
	Reverse	CGATCCGAGGGCCTCACTA
IL-1β	Forward	TGCCACCTTTTGACAGTGATG
	Reverse	TGATGTGCTGCTGCGAGATT
IL-6	Forward	AGACAAAGCCAGAGTCCTTCAG
	Reverse	TGTGACTCCAGCTTATCTCTTGG
TNF-α	Forward	TAGCCCACGTCGTAGCAAAC
	Reverse	GCAGCCTTGTCCCTTGAAGA
Pik3ca	Forward	AGGCTCAGGCACTATTCCATT
	Reverse	TGTGCTGTCTCAACTACGGA
220 (homo)	Forward	ATGGCTCAGTGGGTAAGAGC
	Reverse	CCATGTGGTTGCTGGGATTTG
U6	Forward	GCTTCGGCAGCACATATACTAAAAT
	Reverse	CGCTTCACGAATTTGCGTGTCAT
β-actin	Forward	TATAAAACCCGGCGGCGCA
	Reverse	GTCATCCATGGCGAACTGGTG

Table S2 Primer sequences of RNA pull-down

Primer	Sequence
220-F	AGAGACAAGGAAACAGGCTCTGA
220-R	AAAAGATAAGTAAAGCCAGGGGGTGG
