# Supplementary figures and images for: LncRNA 220: A Novel Long Non-Coding RNA Regulates Autophagy and Apoptosis in Kupffer Cells via the miR-5101/PI3K/AKT/mTOR Axis in LPS-Induced Endotoxemic Liver Injury in Mice
Source: Int J Mol Sci. 2023 Jul 7;24(13):11210. doi: 10.3390/ijms241311210 (PMC10342868; doi:10.3390/ijms241311210)

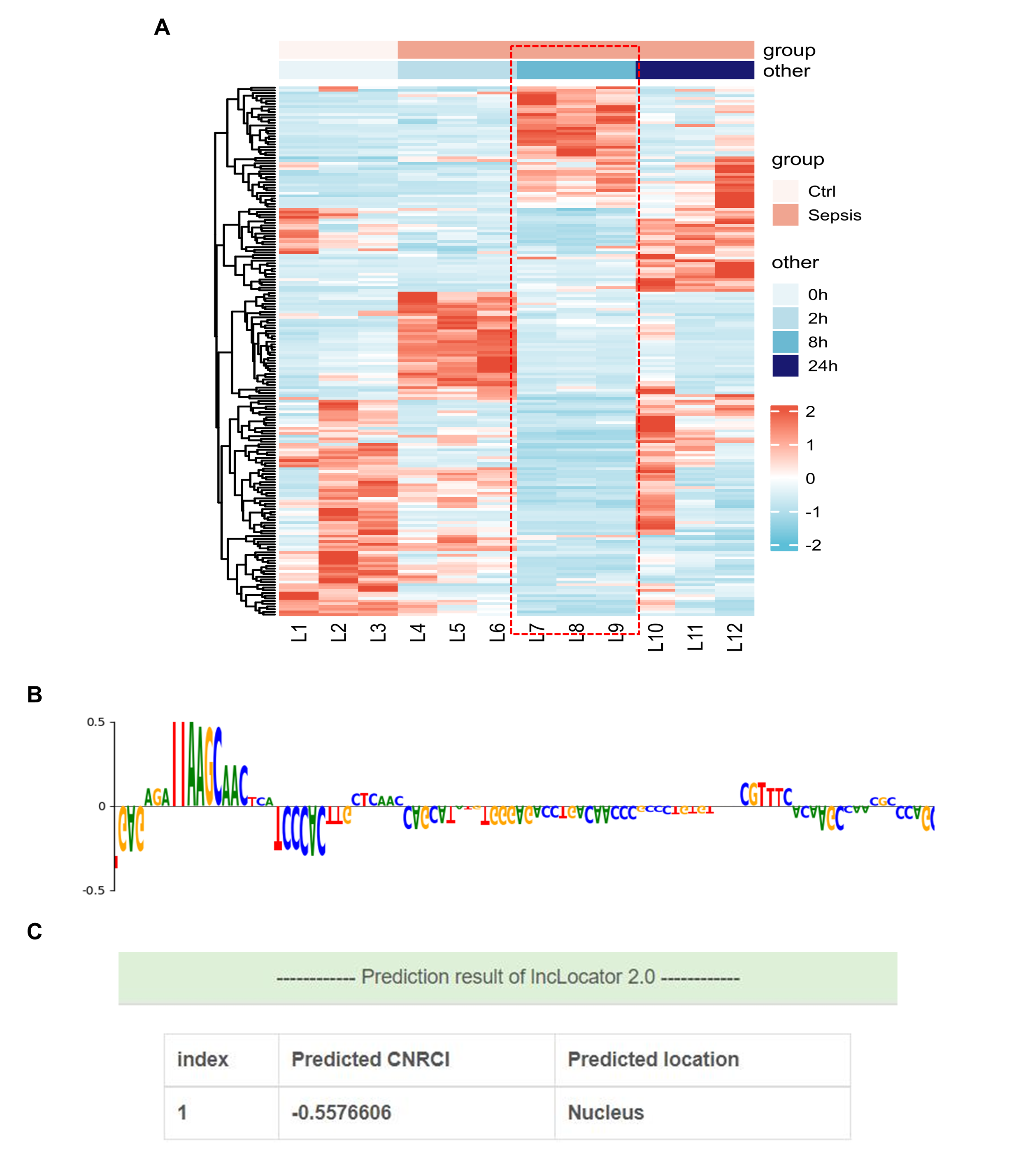

Supplement: Supplementary file 1 [file ijms-24-11210-s001.zip › Supplementary Figure(6.9)/Figure S1.TIF]

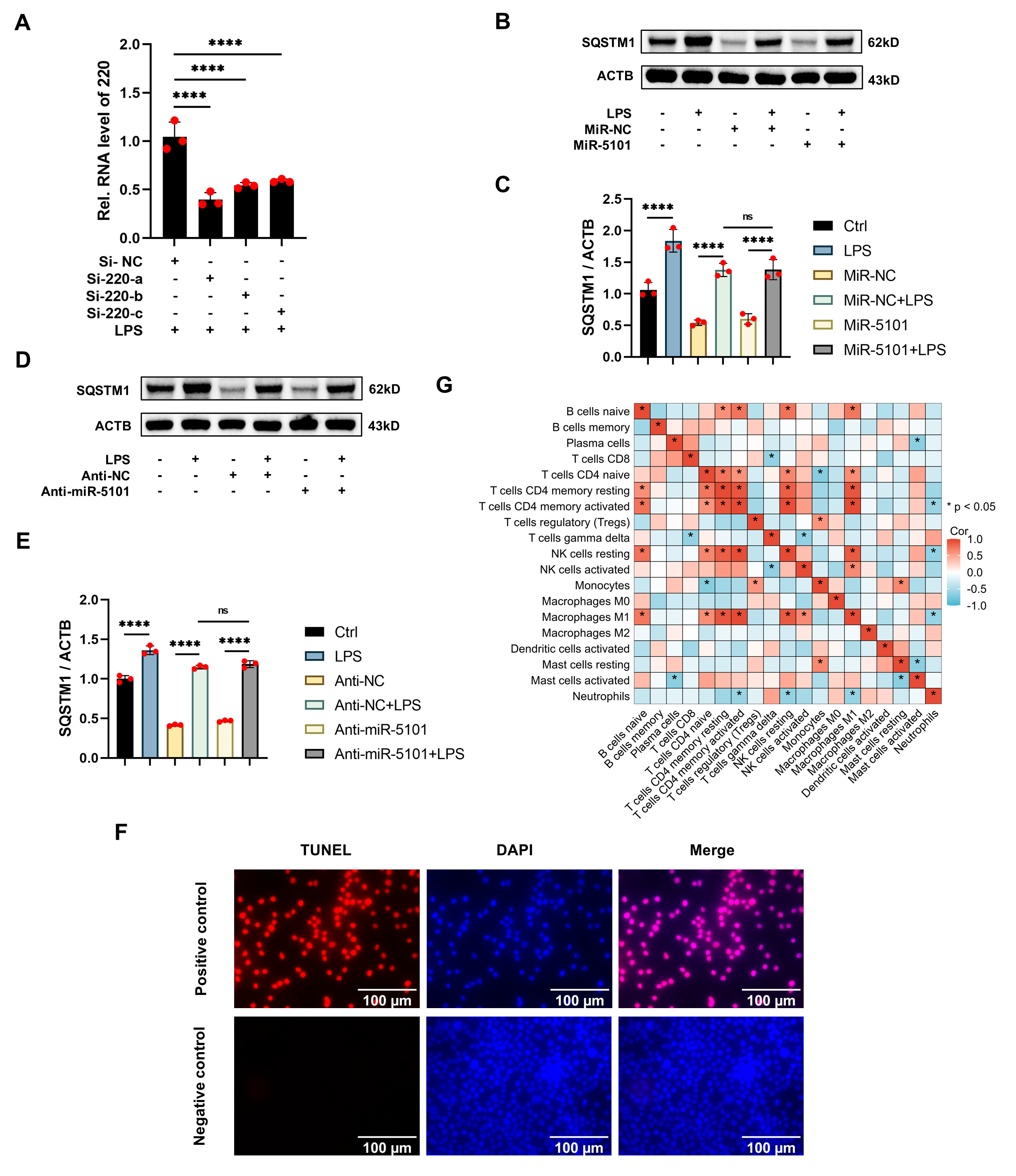

Supplement: Supplementary file 1 [file ijms-24-11210-s001.zip › Supplementary Figure(6.9)/Figure S2.TIF]
